# Mucosal leishmaniasis: epidemiological and clinical aspects

**DOI:** 10.1016/S1808-8694(15)31181-2

**Published:** 2015-10-19

**Authors:** Marcus Miranda Lessa, Hélio Andrade Lessa, Thomas W.N. Castro, Adja Oliveira, Albert Scherifer, Paulo Machado, Edgar M. Carvalho

**Affiliations:** 1 Associate researcher in the immunology and otorhinolaryngology units of the HUPES at the Federal University of Bahia. Doctor of sciences in otorhinolaryngology at FMUSP. Fellow in nasosinusal endoscopic surgery, Graz university, Austria; 2 Adjunct professor of otorhinolaryngology of the Medical School of the Federal University of Bahia. Head of the otorhinolaryngology unit of the HUPES; 3 Medical resident in the otorhinolaryngology unit of the HUPES Federal University of Bahia; 4 Medical resident in the otorhinolaryngology unit of the HUPES Federal University of Bahia; 5 Adjunct professor of the biointeraction department of the Health Science Institute of the Federal University of Bahia; 6 Associate researcher in the immunology unit of the HUPES at the Federal University of Bahia; 7 Full professor in the internal medicine department of the Medical School of the Federal University of Bahia. Head of the immunology unit of the HUPES

**Keywords:** granulomatous disease, leishmaniasis, mucosal leishmaniasis

## Abstract

Leishmaniasis has been documented in several countries, with an estimated prevalence of 12 million people and an incidence at around 400,000 new cases per year. Leishmaniasis in the New World is one the major endemic diseases in Brazil and Latin America. **Objective**: The aim of this study was to add to the current knowdlegde on mucosal leishmaniasis, bringing the experience of the Imunology and Otolaryngology Departments in the Professor Edgar Santos University Hospital of the Federal University of Bahia.

**Conclusion:**

Cutaneous leishmaniasis is the most common form of New World Leishmaniasis; mucosal legions may occur simultaneously or after years of disease. Mucosal leishmaniasis is caused mainly by L. braziliensis braziliensis; although the nasal mucosa is the most affected area, lesions may be found on the lips, mouth, pharynx and larynx. In addition to parasite-related factors, the host immune response may be involved in the pathogenicity of lesions in mucosal leishmaniasis.

## INTRODUCTION

Studies on leishmaniasis in the Southwestern portion of the state of Bahia started in the 1970s. A leishmaniasis unit was set up in the Três Braços region, in the town of Wenceslau Guimarães.^1^ Most of the cases of leishmaniasis diagnosed in 20 municipalities are referred to the health unit that was built in Corte de Pedra, a village located 30 km from the Três Braços region. Currently, about 500 cases of cutaneous leishmaniasis and 10 to 20 cases of mucosal leishmaniasis are seen at this health unit each year. In this paper we reviewed the epidemiology and clinical findings of mucosal leishmaniasis, based on the experience of the Immunology and Otorhinolaryngological Units of the Professor Edgar Santos University Hospital, which is part of the Federal University of Bahia.

## EPIDEMIOLOGY AND TAXONOMY

The term leishmaniasis refers to the infection of vertebrate hosts by protozoans of the genus Leishmania, which belongs to the order Kinetoplastida. Similar to other trypanosomes, they typically present extranuclear DNA in their cytoplasm within a mitochondrial organelle called the kinetoplast. This genus features two developmental forms during its biological cycle in host organisms, as follows: amastigotes, obligatory intracellular parasite in vertebrates, and promastigotes, which develop in the intestine of invertebrate vectors or in axenic cultures.

Leishmanioses are primarily zoonotic infections that affect species other than Man, which may become involved secondarily; transmission between humans may occur and may even predominate or become exclusive. Transmission is usually due to the bites of sand flies in the genuses Phlebotomus and Lutzomya, depending on geography.

American tegumentary leishmaniasis (ATL) is among the major endemic diseases in Brazil and Latin América.^2^ The growing number of new cases is evident, even when taking into account failures in compulsory reporting. A calculation has suggested that the worldwide prevalence of leishmaniasis is 12 million people in 80 countries; estimates suggest that there are 400,000 new cases each year.

Leishmaniases are widely distributed and have been documented in Africa, Europe, Asia and America.^3^ In the Old World the main causal agents of this disease are L. Tropica, L. major and L. aethiopica, which cause tegumentary leishmaniasis, and L. donovani and L. infantum, which cause visceral leishmaniasis. In the Americas, L. chagasi is associated with visceral leishmaniasis; various leishmania species are able to produce tegumentary leishmaniasis, such as: L. braziliensis (Lvb), L. amazonensis, L. guianensis, L. pananmensis and L. mexicana. The characterization of leishmania species, which was initially done based on the behavior of the parasite in the vector, today is based on biochemical, immunological and molecular biological techniques. These include isoenzyme analysis, monoclonal antibody reactivity and kinetoplast DNA analysis.^4^

Mucosal leishmaniasis is a form of tegumentary leishmaniasis that is associated with L. braziliensis, L. panamensis and less frequently with L. amazonensis.

Prior studies have brought attention to the risk factors for developing mucosal leishmaniasis, namely the presence of lesions above the pelvic girdle, large cutaneous ulcers and inadequately treated cutaneous leishmaniasis.^5^ Isolated series have also demonstrated a high frequency of mucosal disease in disseminated cutaneous leishmaniasis patients.^6^ Specifically in such cases, there are multiple lesions both below and above the pelvic girdle, including significant involvement of the face.^6^

## CLINICAL FINDINGS

**Figure 1:**
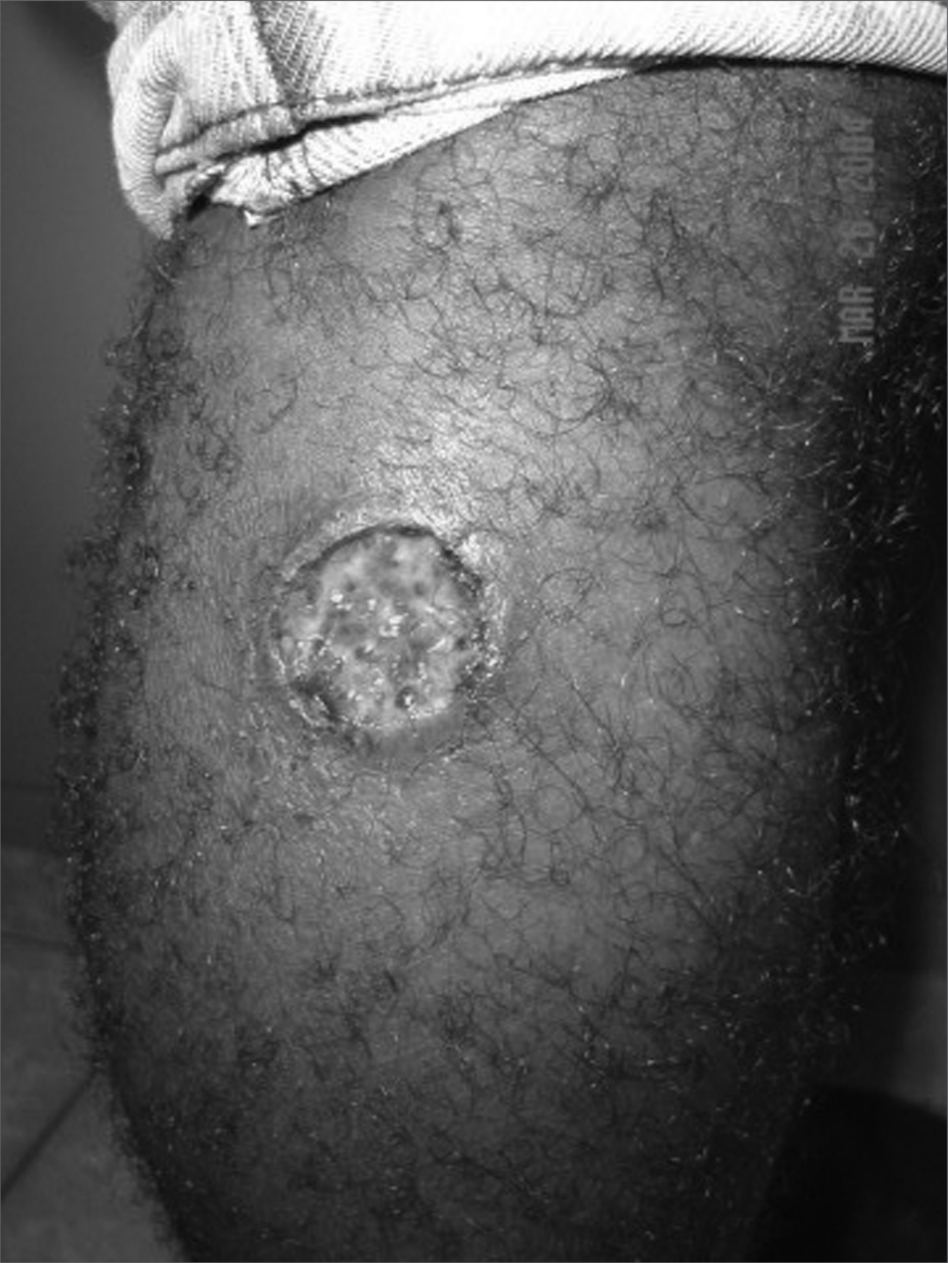
Tegumentary lesion in the right lower limb, showing a welldefined ulcer with elevated borders.

The frequency of tegumentary leishmaniasis is higher compared to visceral leishmaniasis. Classically it is characterized by well-defined ulcers with elevated borders (Figure 1). In ATL caused by L. braziliensis, recent studies have shown that lymph nodes close to the parasite inoculation site are enlarged days or weeks before the lesions appear. Lymph node enlargement is painless or mildly painful and is commonly documented at over 5 cm in diameter. As most of the ulcers caused by L. braziliensis are located in the lower limbs, most of these lymph nodes are found in the groin, the crural region, and less frequently in the popliteal region. In the upper limbs epitrochlear and axillary lymph nodes may be involved. Lymph node enlargement regresses after a few weeks, although it may still be found in patients with cutaneous ulcers lasting over 2 months.

In L. brasiliensis transmission regions around 3% of cutaneous leishmaniasis patients will concomitantly or after disease remission develop a mucosal form of the disease, known as espundia. Two other clinical forms of tegumentary leishmaniasis are well known besides the classical cutaneous and the mucosal forms: disseminated cutaneous leishmaniasis and diffuse cutaneous leishmaniasis. Both forms present multiple lesions, but there are clear clinical, histological, pathological and immunological differences in these conditions.^8^ In Brazil diffuse cutaneous leishmaniasis is caused by L. amazonensis and presents as nodules. The intradermal test using leishmania antigens is negative as there is no cellular immune response to parasites. Histopathology is characterized by a macrophage infiltrate full of leishmania.^6^ Disseminated cutaneous leishmaniasis presents acneiform lesions that eventually may become ulcers. Histopathology reveals an eosinophilic and/or lymphoplasmocytic infiltrate; parasites may or may not be found in the lesion. The immune response is variable, but is preserved in most cases.6 In diffuse cutaneous leishmaniasis the nasal mucosa is not involved; in the disseminated form, involvement of the nose is frequent.^6^

The reason why leishmaniasis patients develop mucosal involvement is not fully clear. The association between L. braziliensis and this form of the disease suggests that parasite-related factors, as well as host conditions, may play a relevant role in mucosal involvement. It is currently accepted that clinically evident leishmaniasis depends on parasite factors, host resistance and the level of the immunological response.^9^ Progression of the disease in Man, from cutaneous to nose lesions, appears to take place through the lymphatic system, blood vessels (metastases) and rarely by direct contact between the mucosa and the cutaneous lesion, such as in the case of a mother with a cutaneous lesion close to the nipples whose newborn develops a mucosal lesion in the mouth. It is also known that vessels in the anterior nasal septum (Kiesselbach's zone) provide good conditions for the development of amastigotes. According to certain authors, lower temperatures in the nose and the passageways for food make it easier for parasites to install themselves in these regions.^10^ The association between lower temperatures and leishmania parasites may in part be explained by in vitro experiments showing that macrophages cultivated at 29°C are less able to destroy parasites compared to macrophages cultivated at 33°C.^10^

**Figure 2:**
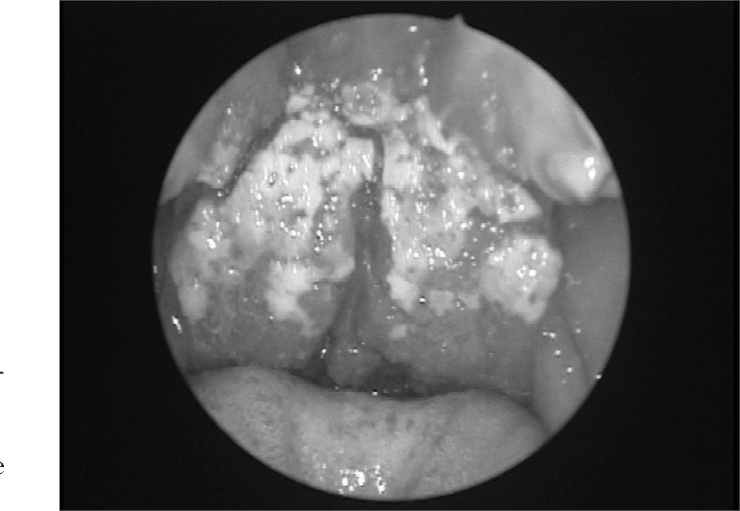
Endoscopy of the right nasal fossa showing perforation of the anterior nasal septum (*) and a granulomatous lesion involving the mucosa in the anterior nasal septum, the tip of the lower turbinate and the floor of the nasal fossa (CI = lower turbinate; Se = septum).

In 1936 Barbosa noted the importance of mucosal leishmaniasis based on case studies in the otorhinolaryngology unit of the Santa Casa de Misericordia Hospital in Sao Paulo.^11^ The nose mucosa is one of the preferred sites for L. braziliensis to cause lesions. Although the mouth, the pharynx and the larynx may be involved, the favorite sites for manifestations of this disease are the cartilaginous nasal septum and anterior portions of the nasal fossae such as the vestibule, the lower turbinates and the floor of the nose (Figure 2). Mucosal involvement is usually secondary to cutaneous lesions, although there are cases where the mucosa is the primary site. In over 90% of mucosal lesions - whether primary or secondary - the nose is the only site with disease.^8^

Both cutaneous and mucosal leishmaniasis predominate in male adults,^5^,^12^ although recently more children of both sexes are being documented with the disease.^13^

Certain patients may present nasal mucosa involvement in the absence of cutaneous disease.^12^ Although data on the penetration site of leishmania in these cases may be absent, and subclinical forms of the disease may not be demonstrated, it is possible that parasites may have entered at the limen nasi - the cutaneous-mucosal transition of the nose - in patients with mucosal disease and no cutaneous involvement.

Studies undertaken in the Três Braços (state of Bahia) endemic region have shown that mucosal leishmaniasis may present up to 264 days following a cutaneous lesion. Only 7% of patients, however, developed mucosal disease after over 10 years after the presentation of a cutaneous lesion.^8^

**Figure 3:**
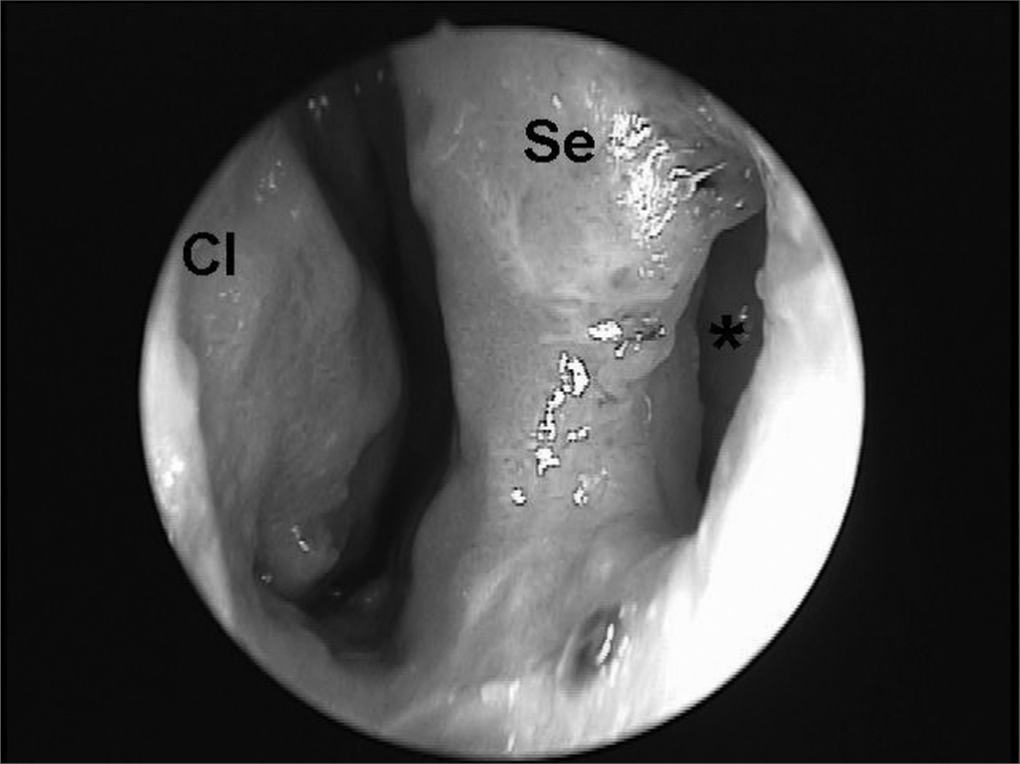
Deformity of the external structure of the nasal pyramid in a patient with mucosal leishmaniasis.

The earliest symptoms and signs of mucosal leishmaniasis are nose block, nose bleeding and granulomas in the anterior nasal septum.^8^ Initially, the anterior septal mucosa is warm and swollen and nodules eventually develop. During this phase patients present rhinorrhea, eventually presenting septal perforation after a few days or months. The skin of the nose may be thickened, swollen and hyperemic, which increases the volume of the nasal pyramid. As the disease progresses, patients begin to present a leishmaniotic facies known as the “tapir nose”, which results from edema of the lining and supporting structures of the nose. Eventually, aggression of tissues may involve the whole nose, the upper lip, the palate and the pharynx, resulting in severe deformity (Figure 3), mutilation, and feeding, breathing and phonation difficulties.

The differential diagnosis includes other granulomatous diseases such as blastomycosis (Paracoccidiose braziliensis), leprosy and tuberculosis, all of which may also present in the anterior nasal septum. The connective tissue reaction in L. braziliensis infection is significant. Scarring characterized by whitish cords during the healing of mucosal lesions separates leishmaniasis from other anterior nasal granulomas.

## INVOLVEMENT OF THE MOUTH, PHARYNX AND LARYNX IN MUCOSAL LEISHMANIASIS

**Figure 4:**
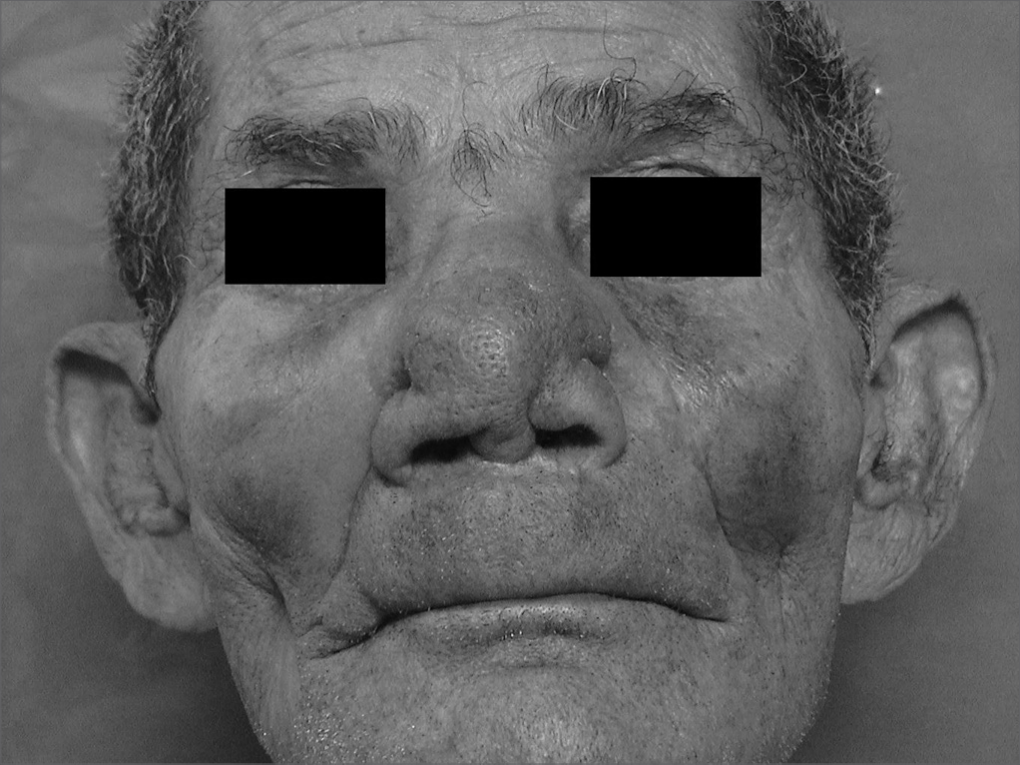
Vegetating granulation tissue involving the soft palate and the uvula, covered with a tenuous fibrin layer.

The pharynx is the second preferred site for mucosal lesions caused by Leishmania braziliensis. Similar to the nose, a mucosal lesion in the pharynx takes on a melon-like quality; however the edema is significantly increased, particularly in the uvula, the tonsillar pillars and the posterior wall of the pharynx. Granulation tissue, that permeates the mucosal findings, is not extensive in this phase. The next phase witnesses the increase of granulation tissue, which significantly destroys tissue; it also involves the lymphoid tissue in Waldeyer's lymphatic ring in the tonsillar region. Areas with a tenuous fibrin layer mix with granulation tissues, with its vegetating appearance (Figure 4). Abundant fibrous tissue may be found, forming true whitish cords that deform completely the anatomical structures of the palate and posterior pharynx due to the intense tissue aggression during the specific post-treatment healing process; this process results in significant stenosis in the transition between the oropharynx and the rhinopharynx.^14^

The laryngeal mucosa is the third preferred site for mucosal involvement in leishmaniasis. Similar to the pharynx, mucosal lesions present the same type of fine granulous tissue. With increased inflammation, vegetating granulous tissue covered with a tenuous fibrin layer involves the mucosa, the epiglottis, and extends to the mucosa of the laryngeal vestibule and the vocal folds. During this phase dysphonia - characterized by a muffled voice - is always present, which brings attention to the vocal folds.^14^

Involvement of the cartilage in the epiglottis and the arytenoids may occur as in the cartilaginous septum to a greater or lesser degree, resulting in perichondritis, which makes swallowing very painful. Painful dysphagia in varying degrees hinders normal feeding, leading to malnutrition and even cachexia. Cartilaginous deformity is significantly evident in post-treatment healing; fibrous tissue is whitish and completely changes the anatomy, resulting in permanent residual dysphonia.^14^

The ears are usually not involved in mucosal leishmaniasis. Mucosal involvement of the rhinopharynx, however, affects the pharyngeal orifice of the Eustachian tube. In these cases otitis media with effusion may be present (chronic secreting otitis media); this finding was present in three cases in our series.^15^ The feeling of a blocked ear, tinnitus and dysacusis are the complaints found in such cases.

Mucosal leishmaniasis may involve the mucosa of the lips and the gingival border. This is a less common presentation of the disease. Under these conditions a differential diagnosis should be made with blastomycosis, particularly due to involvement of the gingival mucosa, a frequent finding in Paracoccidioidis braziliensis infection.

## FINAL COMMENTS

Mucosal leishmaniasis is a form of tegumentary leishmaniasis that is associated with L. braziliensis, L. panamensis and less frequently with L. amazonensis. The frequency of this disease varies in epidemiological and clinical studies. Although the nose mucosa is the preferred site for the mucosal form of leishmaniasis, other sites may include the lips, the mouth, the pharynx and the larynx. This conditions results in high morbidity. The middle ear may be involved in more advanced forms of mucosal leishmaniasis (chronic otitis media with effusion) due to granulomatous tissue in the rhinopharynx, which alters the function of the Eustachian tube. The differential diagnosis of mucosal leishmaniasis includes other granulomatous diseases such as blastomycosis (Paracoccidiose braziliensis), leprosy and tuberculosis, all of which may also present in the anterior nasal septum.
